# Soft Microdenticles on Artificial Octopus Sucker Enable Extraordinary Adaptability and Wet Adhesion on Diverse Nonflat Surfaces

**DOI:** 10.1002/advs.202202978

**Published:** 2022-08-17

**Authors:** Gui Won Hwang, Heon Joon Lee, Da Wan Kim, Tae‐Heon Yang, Changhyun Pang

**Affiliations:** ^1^ School of Chemical Engineering Sungkyunkwan University (SKKU) 2066 Seobu‐ro, Jangan‐gu Suwon Gyeonggi‐do 16419 Republic of Korea; ^2^ School of Electronic and Electrical Engineering Sungkyunkwan University (SKKU) 2066 Seobu‐ro, Jangan‐gu Suwon Gyeonggi‐do 16419 Republic of Korea; ^3^ Department of Electronic Engineering Korea National University of Transportation Chungju‐si Chungbuk 27469 Republic of Korea; ^4^ Samsung Advanced Institute for Health Sciences and Technology (SAIHST) Sungkyunkwan University (SKKU) 2066 Seobu‐ro, Jangan‐gu Suwon Gyeonggi‐do 16419 Republic of Korea

**Keywords:** biomimetics, soft microdenticles, switchable adhesion, wet adhesion

## Abstract

Bioinspired soft devices, which possess high adaptability to targeted objects, provide promising solutions for a variety of industrial and medical applications. However, achieving stable and switchable attachment to objects with curved, rough, and irregular surfaces remains difficult, particularly in dry and underwater environments. Here, a highly adaptive soft microstructured switchable adhesion device is presented, which is inspired by the geometric and material characteristics of the tiny denticles on the surface of an octopus sucker. The contact interface of the artificial octopus sucker (AOS) is imprinted with soft, microscale denticles that interact adaptably with highly rough or curved surfaces. Robust and controllable attachment of the AOS with soft microdenticles (AOS‐sm) to dry and wet surfaces with diverse morphologies is achieved, allowing conformal attachment on curved and soft objects with high roughness. In addition, AOS‐sms assembled with an octopus‐arm‐inspired soft actuator demonstrate reliable grasping and the transport of complex polyhedrons, rough objects, and soft, delicate, slippery biological samples.

## Graphical abstract

Inspired by the hierarchical structural and material aspects of an octopus sucker, a soft microstructured switchable adhesive device with a high degree of adaptability is developed. Owing to good adaptability provided by a hierarchical structure, this device can be robustly attached to irregular objects and detach almost immediately upon deflation under both dry and wet conditions.

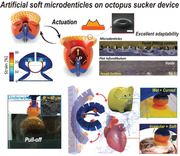

## 1 Introduction

Geometric bending mechanisms inspired by soft finger‐like bodies,^[^ ^1^ ^]^ elephant trunks,^[^ ^2^ ^]^ and octopus arms^[^ ^3^ ^]^ provide adaptive switchable adhesion, which is effective and useful for various applications. Such biologically inspired devices are specialized for picking up a wide variety of curved, rough objects via extreme deformation to completely envelop the objects. However, these systems lack adhesive components (e.g., setae and suction cups), which may inhibit the conformal long‐term attachment of large and morphologically complex objects in both dry and underwater environments. Although intelligent adhesives that mimic gecko feet,^[^ ^4^ ^]^ tree frogs,^[^^]^ and beetle toepads^[^ ^6^ ^]^ remain effective attachment strategies, these systems are limited to dry environments because their adhesive mechanisms (e.g., shear friction and van der Waals interactions) are severely weakened upon contact with water. Very recently, smart materials and devices capable of reversible adhesion in water have been developed by simulating aquatic organisms (octopus, remora suckerfish, and bloodworm).^[^ ^7^ ^]^ However, to mimic the ability of natural animals, further investigation of hierarchically structured adhesive devices with extraordinary adaptability to objects with rough or soft surfaces is required.

The switchable attachment system of *Octopus vulgaris* is a fascinating and appealing model for soft functional materials and applications in dry and wet/underwater environments.^[^ ^8^ ^]^ The octopus can approach an object using its freely deformable arms that are densely populated with suction cups.^[^ ^9^ ^]^ It has been hypothesized by zoologists that the sucker aligns the infundibulum with the targeted object, and upon contact, the hierarchically structured, microscale denticles assist in form‐fitting attachment and sealing of the sucker to the surface of the object.^[^ ^9^ ^]^ The dynamics of the acetabulum located inside the suction cup manipulate the pressure difference between the interior and exterior of the suction chamber, which is used to generate a suction‐based attachment force for robust adherence to the object. The combined functions of the octopus arm, suckers, and microdenticles provide switchable, robust, and conformable attachment and detachment capabilities with various objects in dry, wet, or underwater conditions. However, a methodological interpretation of the degree of adaptability achieved by these intriguing microdenticles is yet to be realized.

Based on enhanced adhesion interaction mechanisms (e.g., suction force and capillary interaction) that are effective in wet/water and dry environments, several bioinspired soft devices have been developed.^[^ ^10^ ^]^ A variety of advanced materials and systems have been proposed that demonstrate switchable adhesion capabilities using diverse actuating methods (e.g., negative pressure and electric fields).^[^ ^4^, ^11^ ^]^ For example, a biorobotic disk inspired by remora suckerfish yielded robust attachment on planar and highly rough surfaces underwater by inducing a pressure difference within the disk upon actuation of its lamellae.^[^ ^7b,c^ ^]^ Nevertheless, owing to its planar adhesive disk, the adhesive capabilities of this suckerfish‐like soft robot are more suited to controllable locomotion than to adaptable attachment to highly curved and deformable objects. To date, various octopus‐inspired soft devices with switchable attachment capabilities have been reported.^[^ ^7^, ^12^ ^]^ However, the actuation mechanisms of such soft adhesive devices (e.g., magnetic or electro/photoactive mechanisms and vacuum chuck systems) are impractical for use in aqueous environments and difficult to integrate into soft machines and actuators that perform complex functions. A recently developed controllable adhesive device simulates the attachment ability of an octopus sucker by inducing the actuation of a soft dome structure.^[^ ^12a^ ^]^ Nevertheless, owing to the absence of a hierarchical micro/nanoscale architecture at the contact interface, this system cannot achieve switchable adhesion to rough, irregular, or soft surfaces.

Here, inspired by the octopus infundibulum, an adaptable artificial octopus sucker (AOS) device with soft microdenticles (AOS‐sm) is developed for effective switchable adhesion on diverse nonflat or soft surfaces. The adhesion performance of the AOS‐sm device is determined by the expansion of the internal void dome and interfacial interactions between the object surface and the infundibulum. AOS‐sm achieves attachment forces of ≈29 and ≈49 N with dry and wet flat substrates, respectively. In particular, the soft microdenticle structure shows high adaptability and conformal contact with rough surfaces. On very rough substrates (*R_a_* = 200 µm), AOS‐sm exhibits robust adhesion (≈17 N), which is three times stronger than that achieved by other AOS devices with a soft or hard flat infundibulum. Further, an soft gripper like octopus (octo‐gripper) is fabricated by assembling an octopus‐arm‐inspired soft actuator (OASA) with AOS‐sms to transport complex polyhedrons, rough objects, and delicate, wet biological samples. (e.g., pig heart, and liver). This conformal octo‐gripper, which exhibits new mechanisms for simulating the biological attachment of octopuses in dry and wet environments, is likely to find application in industrial robots, exploration in harsh environments, and advanced minimally invasive medical technologies (e.g., surgical, implant, and drug‐delivery robots).

## 2 Results and Discussion

### 2.1 Bioinspired Design of AOS‐sm and OASA

**Figure 1:**
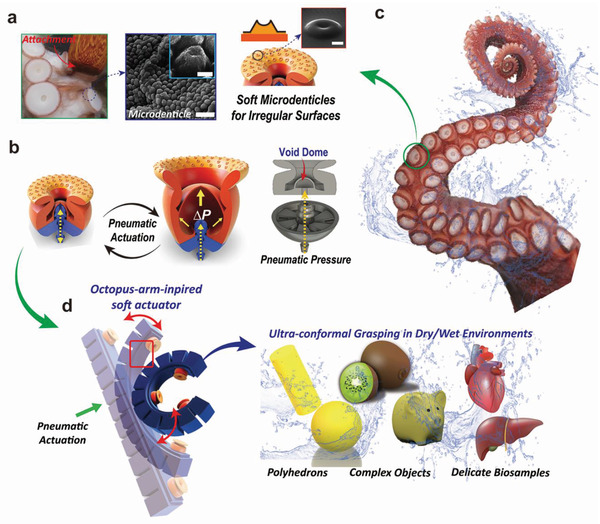
Bioinspired microdenticle soft actuator with versatile controllable adhesion. a) Attachment of an octopus sucker with microdenticles on its infundibulum (scale bar, 10 mm; inset scale bar, 2 mm) and schematic of highly soft microdenticles imprinted on the AOS‐sm infundibulum for enhanced attachment on irregular surfaces (inset: scanning electron microscopy (SEM) image of a single microdenticle; scale bar, 10 mm). b) Schematic of an AOS‐sm that undergo pneumatic actuation to expand its inner chamber. c) Tentacle of *Octopus vulgaris* arrayed with numerous suckers. d) Schematic of a soft OASA integrating multiple AOS‐sms that undergoes pneumatic actuation for conformal attachment on complex polyhedrons, rough objects, and delicate biological samples in dry and wet environments.

As shown in **Figure** **1**a, AOS‐sm with high adaptability to surfaces with irregular morphologies was fabricated by mimicking an octopus sucker. AOS‐sm has a hierarchical structure similar to that of the microdenticle of an octopus sucker that forms a contact surface with an object. The microdenticle of the octopus can enhance interfacial interactions with the surfaces of various objects,^[^ ^9b,c^ ^]^ and the hierarchical structure in AOS‐sm shows a similar effect. The hierarchical structure of AOS‐sm is implemented using a relatively soft material (*E* ≈ 10 kPa)^[^ ^12a^ ^]^ that shows suitable adaptability to various surfaces. In addition, numerous microdenticles enhance the interactions with irregular interfaces through suction and capillary effects.^[^ ^13^ ^]^ As shown in Figure 1b, AOS‐sm is combined with a 3D‐printed structure that applies pneumatic pressure. The suction‐based attachment force is controlled by inducing a structural change in AOS‐sm through pneumatic actuation (0–80 kPa). The octopus that inspired AOS‐sm has numerous suckers on one arm, as shown in Figure 1c. Prior to object attachment, an octopus achieves conformal contact between its suckers and the object through arm movements. Inspired by this, an octo‐gripper was fabricated by connecting three AOS‐sms to an OASA, as shown in Figure 1d. This integration with the OASA allows the AOS‐sms to approach objects from more diverse directions. The adaptive attachment of the octo‐gripper to complex polyhedrons, rough objects, and delicate biological samples in dry and aqueous environments demonstrate the moisture resistance and highly conformal attachment capabilities of this device.

### 2.2 Fabrication of AOS‐sm

**Figure 2:**
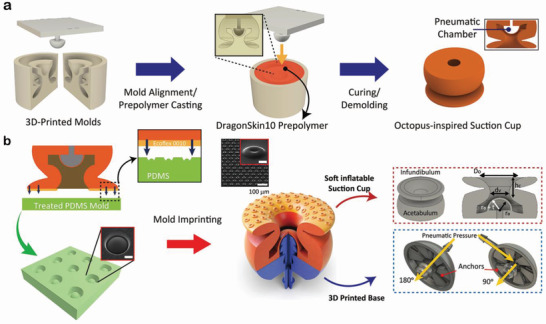
Fabrication and design of AOS‐sm. a) Schematic of the fabrication process for AOS‐sm using molds prepared via 3D printing. b) Schematic of the process for imprinting microdenticles onto the AOS‐SM infundibulum (inset: SEM image of a single microdenticle; scale bar, 10 µm).

The AOS cup (diameter 3.2 cm and height 2 cm) with soft microdenticles was fabricated through a series of facile replica molding processes. According to previously reported methods, the two base molds were sealed together, silicone precursor Dragon Skin 10 (*E* ≈ 300 kPa) was deposited,^[^ ^12a^ ^]^ and then the top mold was attached to form a void dome (**Figure** **2**a). After curing the AOS‐sm body and disassembling from the molds, the microdenticles were replicated by thinly coating the surface of the AOS with an even softer silicone precursor, Ecoflex10 (*E* ≈ 10 kPa),^[^ ^12a^ ^]^ and then applying the AOS to a polyurethane‐acrylate (PUA)‐based mold with the reverse architecture of the microdenticles (Figure 2b and Figure S1, Supporting Information). After curing in an oven at 60 °C for 1 h and disassembling from the PUA mold, the AOS‐sm was combined with a rigid, specially designed 3D‐printed structure that can apply pneumatic pressure, thus allowing the AOS‐sm to adhere to various objects with high adaptability.

### 2.3 Highly Soft Microdenticles of AOS‐sm

**Figure 3:**
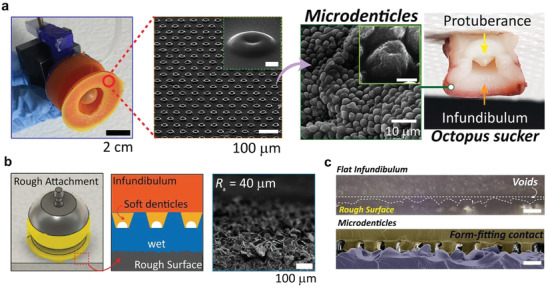
Attachment of AOS‐sm to a rough surface. a) Octopus sucker infundibulum as biological inspiration for microdenticles on the AOS‐sm infundibulum surface (inset scale bar, 2 µm (left) and 10 µm (right)). b) Schematic of the attachment of AOS‐sm on rough surfaces. c) Comparison of SEM images for an AOS with a flat infundibulum and the microdenticles of AOS‐sm on a rough surface (scale bar, 50 µm).

As shown by the SEM image in **Figure** **3**a, the soft microdenticles are densely distributed on AOS‐sm. Furthermore, the geometric properties of these microdenticles are similar to those of the denticules on a natural octopus sucker. To maximize interfacial interactions, a hierarchical structure was designed with a diameter of 30 µm and a spacing ratio of 1; the spacing ratio is the distance between structures divided by the structure width. In several studies, various micro‐sized pillar structures have been optimized.^[^ ^9^, ^13^ ^]^ Figure 3b illustrates the contact process at the interface between AOS‐sm and a rough surface. Cross‐sectional SEM images of a rough substrate (*R_a_* = 40 µm) demonstrate that the conformal contact provided by the soft microdenticles on AOS‐sm is superior to that achieved by an AOS with a flat infundibulum (Figure 3c). Some voids that cannot be filled are present at the interface between the smooth, flat infundibulum and the rough substrate owing to a lack of adaptability. In contrast, the soft microdenticles with high adaptability are in conformal contact with the rough substrate.

### 2.4 Highly Soft Microdenticles of AOS‐sm on Irregular Surfaces

**Figure 4:**
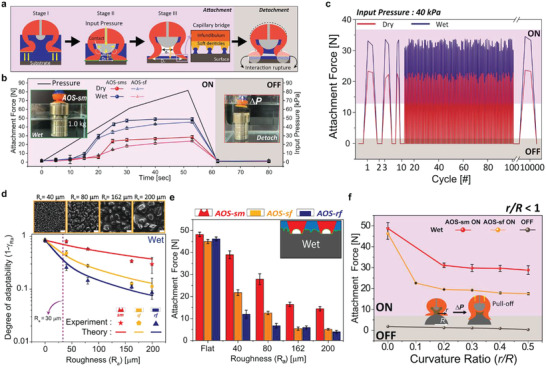
Adhesion performances of AOS‐sm on surfaces with irregular features. a) Schematic of the attachment and detachment mechanism of AOS‐sm under wet conditions. b) Attachment force of AOS‐sm and AOS‐sf on a flat surface under dry and wet conditions as a function of input pressure. c) Cyclic measurements (up to 10 000 times) of AOS‐sm attachment under 40 kPa pneumatic actuation in dry and wet conditions. d) Degree of adaptability profiles of sm, sf, and rf, and SEM images of rough surfaces (*R_a_* = 0, 40, 80, 162, and 200 µm; scale bars, 200 µm). The purple dot line indicates the same *R_a_* as the microdenticle size e) Pull‐off suction‐based attachment force for AOS‐rf, AOS‐sf, and AOS‐sm on surfaces with varying roughnesses under water (inset: schematic of microdenticle interaction with a rough surface underwater). f) Suction‐based attachment force profiles for on (80 kPa) and off (0 kPa) inputs of pneumatic pressure under wet conditions at various AOS‐sm‐to‐surface curvature ratios (*r*/*R*). Error bars represent standard deviations (n = 10).

To estimate the AOS‐sm volume changes according to the input pressure, the changes in the interfacial area and height were measured using a laser displacement sensor and a Vernier scale (Figure S2, Supporting Information). These data were compared with the results obtained using a finite element method (FEM) simulation (Figure S3, Supporting Information). The experimentally observed changes in height and interfacial area were very similar to the simulated values (Figure S4a, Supporting Information). The structural changes of AOS‐sm that generate a suction‐based attachment force can be divided into three stages: 1) Stage I (OFF state), wherein no contact occurs between the inner void dome and the inner wall at pressure inputs of 0–30 kPa; 2) Stage II (contact state), wherein the inner void dome and the inner wall come into contact at a pressure input of 30 kPa; and 3) Stage III (ON state), wherein the internal void dome pushes the inner wall via pneumatic actuation at pressure inputs above 30 kPa (**Figure** **4**a and Figure S5, Supporting Information).

As shown in Figure 4a, during Stage I, AOS‐sm approaches the object by conforming to its surface. During stages II and III, expansion of the void dome changes the interfacial area and height, which increases the chamber volume and leads to a pressure difference between the inner chamber and the environment (Figures S3 and S5, Supporting Information). When the pneumatic is removed from the dome of the AOS‐sm, the seal of the AOS‐sm is easily ruptured for facile and rapid detachment because the vacuum‐assisted attachment force has superiority to the bonding at the interface (Figures S4 and S6, Supporting Information). Based on our investigation, an attachment model can be developed for AOS‐sm using the total suction‐based attachment force (*F_s_*), resulting from both the deformation of the suction cup and the adhesion force of the contact interface of the microdenticle rim in a dry or wet environment. For AOS‐sm under wet conditions, *F_s,w_* is expressed as:

(1) $$F_{s,W}=-\Delta P_{0}\;\left(1-\gamma_{w}\right)\frac{\pi D_{v,in}^{2}}{4}+\sigma_{rim,w}A^{\prime }$$

where Δ*P*_0_ is the pressure difference between the interior and exterior of AOS‐sm (≈ −101.32 kPa), *D_v,in_* is the diameter of the interfacial area, *γ*_*w*_ is a compensation factor which has a value between 0 and 1 owing to seal leakage at the AOS‐sm regions of contact,^[^ ^14^ ^]^ *σ*_*rim*,*w*_ is the capillary interactions induced between the AOS rim and the substrate under wet conditions,^[^ ^13^ ^]^ and *A*′ is the effective interfacial area between the AOS rim–substrate interface. Pressure leakage can be simply explained using a model of the interfacial interaction between the infundibulum of AOS‐sm and the substrate surface.^[^ ^15^ ^]^ This parameter can be described by a linear approximation of the experimental interaction force between the infundibulum and the substrate surface, as shown in Figure S7, Supporting Information (see Supporting Information for detailed derivations and explanations). Accordingly, the degree of adaptability of the infundibulum on a rough surface (1 − *γ*_*Ra*_) can be expressed as:

(2) $$\left(1-\gamma_{Ra}\right)\approx \frac{1-\gamma_{flat}}{\alpha R_{a}+1},$$

where *γ*_*Ra*_ is the compensation factor for a rough substrate with *R_a_*, *α* is the leakage parameter, *R_a_* is the roughness average of the substrate, and *γ*_*flat*_ is the compensation factor for a flat substrate (for further details, see Supporting Information). Therefore, the suction‐based attachment force of AOS‐sm considering the suction‐based attachment force resulting from structural changes ($\approx -\Delta P_{0}\frac{\pi D_{v,in}^{2}}{4}$) and the interfacial interactions ($\approx \frac{1-\gamma_{flat}}{\alpha R_{a}+1}$) between the infundibulum and the surface can be expressed as follows:

(3) $$F_{s,W}\approx -\Delta P_{0}\left(\frac{1-\gamma_{flat}}{\alpha R_{a}+1}\right)\frac{\pi D_{v,in}^{2}}{4}+\sigma_{rim,w}A^{\prime }$$

Figure 4b shows the time‐dependent pull‐off attachment force of AOS‐sm on a flat substrate at variable input pressures. All the suction‐based attachment experiments were performed using a customized apparatus to measure the attachment force under ambient dry and wet conditions (Figure S8, Supporting Information). A 3D‐printed base for AOS‐sm that can be fixed to a jig in the adhesion measurement equipment was designed and used, and pneumatic pressure was applied to the void dome at increments of 10 kPa. The observed attachment force behavior can be explained by the pressure changes inside the suction chamber owing to structural changes and the interfacial interactions between the infundibulum and the object surface. The pressure change in the AOS‐sm chamber was measured using a custom device and compared to the theoretical value (Figure S9, Supporting Information). The experimental pressure differences in wet and dry environments (≈ 86 and ≈50 kPa, respectively) were consistent with the theoretical values considering the volumetric changes and pressure leakage (Figure S4b,c, Supporting Information). In a dry environment, the adhesion interactions of microdenticles are induced by van der Waals interactions (see Supporting information for details). Based on our investigation, it was confirmed that the interaction between the AOS rim and the contact substrate contributed to the attachment force of the AOS‐sm in both dry and wet conditions. The interaction between the AOS rim and the contact substrate contributed to the attachment force of the AOS‐sm in both dry and wet conditions (Figures S6 and S10, Supporting Information). Interestingly, the contribution of the microstructured rim to the AOS‐sm adhesion decreases as the contact area (*A*′) of the interface decreases as the input pressure increases in the AOS‐sm (Figures S4d, S5, and S6, Supporting Information).

As shown in Figure S10, Supporting Information, the theoretical values in a dry or wet environment well agree with the experimental adhesion forces of AOS‐sm. Here, a maximum dry suction‐based attachment force of up to ≈29 N and a maximum wet suction‐based attachment force (*F*_*s*,*W*_) of ≈49 N at an input pressure of 80 kPa (Figure 4b and Video S1, Supporting Information). Interestingly, the AOS‐sm and AOS with a soft flat rim surface (AOS‐sf) suction‐based attachment force for dry/wet conditions increase negligibly for relatively higher input pressures (40–80 kPa). This is because according to simulations in Figures S3 and S4, Supporting Information, geometric changes of the AOS‐sm as well as pressure difference inside/outside the AOS‐sm chamber are saturated due to the characteristics of their elasticity. In comparison to AOS‐sm, the AOS‐sf demonstrated lower suction‐based attachment force in both dry/wet conditions for each input pressure. To verify the durability of the suction‐based attachment force of AOS‐sm, the input pressure (≈40 kPa) was changed periodically to test the attachment force in cyclic ON/OFF states (Figure 4c). AOS‐sm exhibited robust, reliable, and repeatable attachments up to 10 000 times in the ON state for both dry and wet environments and high responsiveness with instant release in the OFF state. Table S1, Supporting Information compares the attachment/adhesion force per unit area of our AOS‐sm to other methods described in former literature. While AOS‐sm is capable of both dry and wet attachment reliably, most previously developed bioinspired adhesive actuators can achieve only one of either dry/wet attachment due to limitations of their actuation methods or the mechanisms of achieving adhesion to a surface in a specific environment.

As shown in Figure 4d and Figure S11, Supporting Information, the degree of adaptability was measured to demonstrate the interfacial interaction force of the infundibulum with soft microdenticles (sm) against various rough surfaces (*R_a_* = 40, 80, 162, and 200 µm). Herein, *R_a_* is the deviation of a surface from a mean height, wherein a higher *R_a_* value indicates a rougher and more uneven morphology. The degree of adaptability of sm to various rough surfaces (*R_a_* = 40, 80, 162, and 200 µm) under wet conditions is relatively high compared to those of a soft flat infundibulum (sf) and a rigid flat infundibulum (rf). To verify the benefits of the soft microdenticles, the suction‐based attachment force of AOS‐sm was compared to those of AOS samples coated with a soft flat surface (AOS‐sf) and a rigid flat surface (AOS‐rf) at the maximum pneumatic pressure (80 kPa) (Figure 4e). As the degrees of adaptability to wet flat substrates are similar, the suction‐based attachment force of AOS‐sm on a flat substrate under wet conditions only shows a slight increase (*F*_*s*,*W* ≈_49 N for AOS‐sm, ≈47 N for AOS‐sf, and ≈48 N for AOS). In contrast, the degree of adaptability of sm to wet rough surfaces (*R_a_* = 40, 80, 162, and 200 µm) is higher than those of sf and rf; therefore, the suction‐based attachment force of AOS‐sm is higher than those of AOS‐sf and AOS‐rf. On a substrate with high roughness (*R_a_* = 200 µm), the attachment of AOS‐sm is approximately three times stronger than that of AOS‐sf and AOS‐rf. The suction‐based attachment force of AOS‐sm on rough surfaces in a wet environment is similar to the theoretical value obtained based on the degree of adaptability of sm (Figure S12, Supporting Information). For AOS‐sm and AOS‐sf, the pull‐off suction‐based attachment forces in dry and wet environments were compared for substrates with various curvatures. Here, the AOS‐sm‐to‐surface curvature ratio is defined as the ratio of the radius of the AOS initial contact region (*r*) to the curvature of the substrate sample to the radius (*R)* of the substrate (Figure 4f and Figure S13, Supporting Information). In AOS‐sm and AOS‐sf, the attachment interface changes owing to structural changes of the infundibulum that occur when pneumatic pressure is applied. When the infundibulum achieves conformal contact with the curved surface, the pressure difference generated in the inner chamber results in a suction effect. When switched to the OFF state, AOS‐sm and AOS‐sf immediately detach from the substrate under both dry and wet conditions because the curvature allows for easy rupture of the seal (Figure 4f and Figure S13, Supporting Information). In both dry and wet environments, the attachment for AOS‐sm is higher than that of AOS‐sf on various curved surfaces owing to the enhanced surface adaptability of the hierarchical microdenticle structure. AOS‐sm is capable of gripping objects of various sizes with *r/R* values ranging from 0 to 0.5.

**Figure 5:**
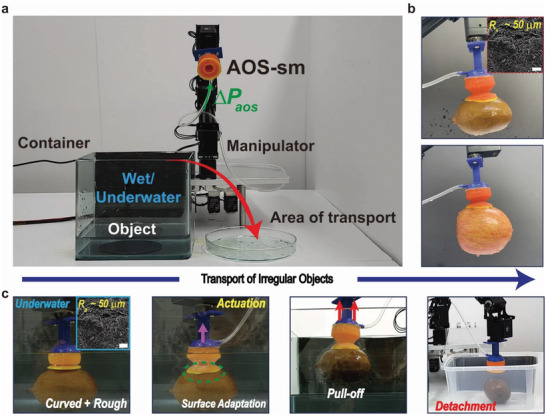
Transportation demonstrations using a single AOS‐sm. a) Experimental setup consisting of an AOS‐sm connected to a manipulator for the transport of various objects under wet conditions. b) Photographs of the AOS‐sm attached to a kiwifruit and an apple. c) Photographs of the transport of a kiwifruit under wet conditions.

As shown in **Figure** **5**a, a single AOS‐sm was integrated with a commercial robot manipulator to demonstrate the attachment and transportation of various rough and curved objects in wet environments. As shown in Figure 5b and Figure S14, Supporting Information, AOS‐sm was able to lift various objects, including a flat, brittle silicon wafer; a relatively heavy (0.5 kg) curved glass bottle of apple juice; a soft, spherical apple; and a kiwifruit with a very rough (*R_a_* ≈ 50 µm, *R_Max_* ≈ 80 µm), curved surface. As shown in Figure 4c, the AOS‐sm establishes remarkably adaptability to the surface of a curved kiwifruit with high roughness (*R_a_* ≈ 50 µm; *R_Max_* ≈ 80 µm). As the AOS‐sm inflates, the infundibulum deforms and adapts to the curvature of the fruit, allowing the microdenticles to establish contact with the rough features. After achieving adaptive contact with the rough and curved surface of a kiwifruit, the AOS‐sm can transfer the kiwifruit to a nearby container and detach almost immediately upon removal of the pneumatic pressure (Video S2, Supporting Information). Moreover, to demonstrate stable and conformal attachment against dynamic surfaces, we used an artificial heart with a dynamic surface of repeated expansion and contraction as the target object (Figure S15 and Video S3, Supporting Information). Here, AOS‐sm can stably hold an artificial heart with a smooth, complex, 3D‐shaped dynamic surface (repetitive expansion and contraction movements).

### 2.5 OASA for Complex Objects and Soft Biosamples

**Figure 6:**
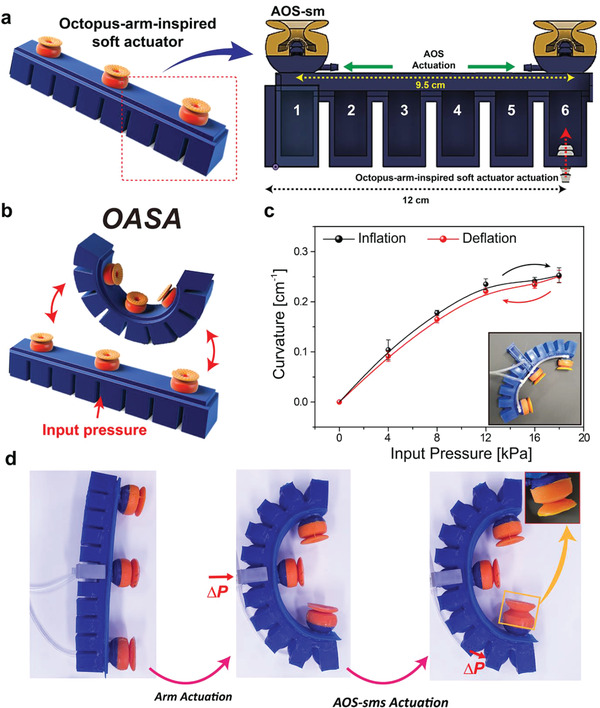
Design and performance of the integrated OASA. a) Schematic of the OASA assembly. b) Schematic of actuation of the OASA. c) Input pressure–curvature profiles for pneumatic inflation (black) and deflation (red) of the OASA. Error bars represent standard deviations (*n* = 10). d) Photographs of the OASA during rest, actuation of the arm, and actuation of the arm and AOS‐sms.

Furthermore, an OASA was assembled by integrating three AOS‐sms with a soft actuator that resembles an octopus‐like arm and deforms responsively to the pneumatic actuation of its inflatable chambers.^[^ ^16^ ^]^ As shown in **Figure** **6**a,b, the OASA (length 24 cm and width 3 cm) has six hollow spaces that are pneumatically inflated and bending. The OASA was fabricated using Dragon Skin 10 (*E* ≈ 300 kPa) and a specifically designed 3D‐printed mold (for details, see Supporting Information and Figure S16, Supporting Information). As illustrated in Figure S17, Supporting Information, signal and pressure circuits were designed to simultaneously regulate the actuation of the OASA arm and the AOS‐sms (for details, see Supporting Information). Initially, the curvature profiles for pneumatic inflation and deflation of the octo‐gripper were measured (Figure 6c). At maximum pneumatic actuation (18 kPa), the octo‐gripper has maximum curvature (≈0.25 cm^−1^). The octo‐gripper and circuits were then connected to a commercial manipulator and controlled using a buttoned module to demonstrate the transport of complex objects (Figure S18, Supporting Information). The OASA arm and AOS‐sm were controlled using different buttons, and the deformations were highly responsive to increases and decreases in input pressure, indicating the ability of the OASA to conform to objects with diverse complexities (Figure 6d).

**Figure 7:**
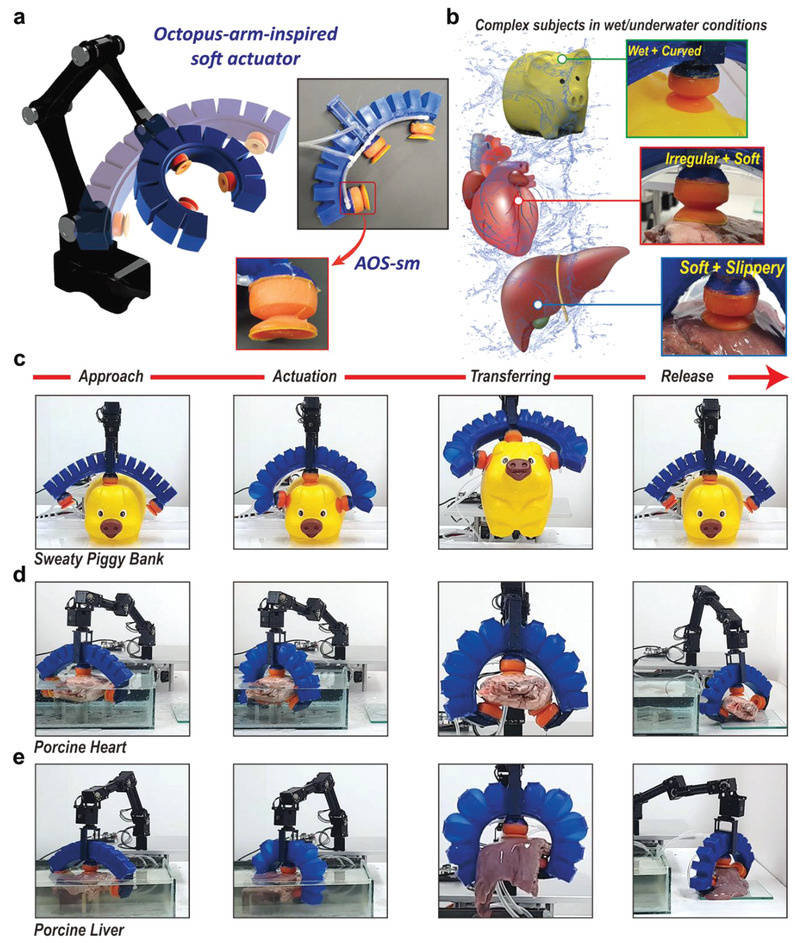
The ability of the OASA to transport wet complex objects. a) Schematic of the OASA assembly with three AOS‐sms and a soft inflatable arm (insets: photographs of the actuated OASA and a single AOS‐sm). b) Illustrations of “sweaty” piggy bank, porcine liver, and heart (insets: photographs of an AOS‐sm attaching conformably to the irregular surface of each object). c) Photographs of the OASA manipulating a piggy bank under “sweaty” conditions. d) Photographs of the OASA transferring a porcine heart to a glass dish under wet conditions. e) Photographs of the OASA transferring a porcine liver to a glass dish under wet conditions.

As shown in **Figure** **7**a, the OASA bends in response to the input pressure and the AOS‐sms actuate via a separate pneumatic pressure pathway. The two components are articulated to approach, adapt, and attach robustly to objects. Thus, the octo‐gripper achieves adaptive and highly conformal performance with a “sweaty” piggy bank as well as porcine liver and heart biosamples in a wet environment (Figure 7b and Figure S19, Supporting Information). As shown in Figure 7c, the octo‐gripper first approaches the piggy bank, and the OASA bends to conform to the curvature of this object (Figure 7b and Video S4, Supporting Information). The AOS‐sms then actuate to attach firmly onto the rounded surface of the piggy bank. The OASA can be used to manipulate the piggy bank, allowing long‐term, dynamic handling of the object, followed by facile detachment upon deflation of the OASA and AOS‐sms. The OASA can be approached by bending flexibly according to the characteristics of the big target (see Figure S19, Supporting Information), and the measured suction force can be set to about 29 N in dry conditions and 49 N in wet/water conditions per each AOS‐sm.

As a more advanced application, the facile transport of submerged porcine organs by the OASA was demonstrated (Figure S18 and Video S5, Supporting Information). The heart is highly soft (*E* ≈ 25–200 kPa),^[^ ^17^ ^]^ irregularly shaped,^[^ ^18^ ^]^ and constantly wet owing to fluids within the body. Nevertheless, the octo‐gripper adapts and adheres conformably to the irregularities of the porcine heart (Figure 7d and Video S5, Supporting Information). The octo‐gripper establishes a firm grip underwater, transfers the organ to a glass dish, and then releases it effortlessly upon deflation. Similarly, a porcine liver, which has an even softer (*E* ≈ 5–7 kPa) and more slippery surface than the heart, was successfully transferred (Video S5, Supporting Information).^[^ ^19^ ^]^ As shown in Figure 7e, the actuation of the OASA arm conforms to the complexities of the bulk liver, while the AOS‐sms attach firmly via suction to its highly soft and slippery surface. Upon transfer to the glass dish, the octo‐gripper deflates and detaches from the porcine liver. These demonstrations highlight the highly conformal capabilities of the octo‐gripper as well as its potential for medical applications, including surgical operations.

## 3 Conclusion

We have developed a highly adaptive switchable adhesive device with soft microdenticles based on the hierarchical architecture and muscular actuation mechanism of an octopus sucker. We present a novel mechanism for amplified, multiscale suction‐based attachment force on surfaces with variable roughness features. Soft microdenticles were deposited onto an AOS rim via a facile replica molding technique using a soft silicone elastomer, providing a high degree of adaptability on a rough surface (1 − *γ*_*Ra*_) according to our study. Accordingly, the AOS‐sm exhibited robust attachment to surfaces with varying roughnesses, curvatures, and surface properties, particularly under wet conditions. For practical applications, AOS‐sms were integrated into an OASA, which could firmly and adaptably grasp and transport complex polyhedrons, rough objects, and delicate, wet biological samples. This highly adaptable octo‐gripper is promising for the development of high‐performance bioinspired robotic systems that can be applied in both dry and wet environments. Future research and development, particularly by integrating multiple arrays of AOS‐sm on other soft robotic bodies and devices, would shed light on applications in various fields, such as industrial transport systems, exploration robotics for harsh environments, and next‐generation surgical apparatuses.

## 4 Experimental Section

### Adhesion Measurements of AOS‐sm

All adhesion tests were performed in the direction normal to the flat and curved s‐PUA substrates (area ≈5 × 5 cm^2^) under dry (≈50% relative humidity) and wet conditions using custom equipment (adhesion tester, Neo‐Plus). To perform measurements underwater, the s‐PUA substrate was immersed in distilled water. As shown in Figure S8, Supporting Information, the AOS‐sm sample was fixed on a jig and connected via a tube to an electric pressure calibrator, which was used to ensure the operation of the AOS‐sm according to specific input pressure. The AOS‐sm first contacted the substrate with a negligible preload. When a certain pneumatic pressure was inserted into the void dome, the AOS‐sm was pulled off until the sample was completely detached from the substrate. The adhesion performance with PUA substrates was similar to the adhesion performance with glass or silicon substrates. All measurements were repeated at least 10 times and averaged values are displayed.

### AOS‐sm Chamber Pressure Measurements

The AOS‐sm sample was placed on a 3D‐printed stand, which allowed the AOS‐sm to be operated from the top and the pressure within the AOS‐sm chamber to be measured from the bottom (Figure S9, Supporting Information). The bottom of the AOS‐sm was connected to a pressure sensor (40PC001B1A, Honeywell Inc., USA) using an input tube while applying pneumatic pressure using an electric pressure calibrator. Pneumatic pressures of 0–80 kPa were input into the void dome chamber in 10 kPa increments, and the change in chamber pressure was observed through the difference in voltage output using an oscilloscope (Figure S9, Supporting Information).

### Geometric Measurements of AOS‐sm

Changes in the AOS‐sm chamber height were measured using a compact laser displacement measurement sensor (CD22‐15VM12, Fastus, Japan) attached to an elevated platform (Figure S2, Supporting Information). A laser was placed at the center of the AOS‐sm dome structure using a 3D‐printed model. An electric pressure calibrator (719Pro, Fluke Inc., USA) in the void dome chamber was used to apply pneumatic pressure from 0 to 80 kPa in 10 kPa increments and measure the displacement change. Measurements of the inner (*D*_i_) and outer (*D*_o_) diameters of the AOS‐sm cusp and the diameter of the dilated dome (*d*) were performed using a Vernier scale.

### Statistical Analysis

Data was expressed as mean ± standard deviation (SD). Data plotting was performed by using Origin Pro 2019 statistical software or Microsoft excel.

## Conflict of Interest

The authors declare no conflict of interest.

## Supporting information

Supporting Information

Supplemental Video 1

Supplemental Video 2

Supplemental Video 3

Supplemental Video 4

Supplemental Video 5

## Data Availability

The data that support the findings of this study are available from the corresponding author upon reasonable request.
