# Supplementary material for: Soft Microdenticles on Artificial Octopus Sucker Enable Extraordinary Adaptability and Wet Adhesion on Diverse Nonflat Surfaces
Source: Adv Sci (Weinh). 2022 Aug 17;9(31):2202978. doi: 10.1002/advs.202202978 (PMC9631055; doi:10.1002/advs.202202978)
Supplement: Supplementary file 1 — Supporting Information [file ADVS-9-2202978-s002.pdf]

Supporting Information

Soft Microdenticles on Artificial Octopus Sucker Enable Extraordinary Adaptability and Wet Adhesion on Diverse Nonflat Surfaces

Gui Won Hwang†, Heon Joon Lee†, Da Wan Kim, Tae-Heon Yang, and Changhyun Pang**

Detailed Derivation of AOS-sm Attachment on Substrates Under Dry Conditions

The artificial octopus sucker with soft microdenticles (AOS)-sm first induces contact with a substrate and then undergoes structural changes through pneumatic actuation that produce a suction-based attachment force. The dry attachment force ($F_{s,dry}$), which is the suction-based attachment force of AOS-sm under dry conditions,[1] can be expressed as follows:

$$F_{s,dry} = \frac{P_0(1-\gamma_{dry})\pi {D_v}^2}{4}\left\{1 - \frac{h_0({d_0}^2+d_0D_0+{D_0}^2)}{h_v({d_v}^2+d_vD_v+{D_v}^2)}\right\} + \sigma_{rim,dry}A' \quad \text{(S1)}$$

where P_0 is atmospheric pressure (~101.3 kPa), γ_{dry} is an experimentally determined constant between 0 and 1 that compensates for seal leakage at the AOS-sm regions of contact, D_v is the diameter of the interfacial area when a vacuum is established in the inner chamber, d_v is the diameter of the upper portion of the inner chamber in the vacuum state, h_v is the chamber height under vacuum, d_0 is the diameter of the upper portion of the inner chamber during initial protuberance contact, D_0 is the diameter of the interfacial area of the AOS chamber during this phase, and h_0 is the height of the chamber during this phase. To account the adhesive interactions of the microdenticles, $\sigma_{rim,dry}$ is induced by the van der Waals interactions between the soft microstructured AOS rim and the substrate under dry conditions,[1] and A' is the effective interfacial area between the AOS rim – substrate interface, as shown in Figure S4d. The experimental values of D_v, d_v, d_0, and D_0 were measured using a Vernier scale, whereas h_v and h_0 were measured using a compact laser displacement measurement sensor. The theoretical values of D_v, d_v, d_0, D_0, h_v, and h_0 were obtained from a finite element method (FEM) simulation.

Derivation of AOS-sm Attachment on Substrates Under Wet Conditions

In a wet environment, the suction-based attachment force is obtained by calculating the simple volume change upon deformation and the actual contact area. It can be assumed that there is little internal pneumatic pressure when the internal volume of AOS-sm changes in a wet environment. As the inner pressure is zero, the pressure difference between the interior and exterior of AOS-sm can be maximized, expressed as $\Delta P_{max} = -\Delta P_o$. Hence, the suction-based attachment force of the AOS-sm under wet conditions ($F_{s,W}$) is as follows:

$$F_{s,W} = -\Delta P_0(1-\gamma_w)\frac{\pi D_{v,in}^2}{4} + \sigma_{rim,w}A'. \quad \text{(S2)}$$

Similarly, the experimental and theoretical values of D_v were substituted into Equation (S2) to understand wet adhesive performance.

Derivation of Compensation Factor on Rough Surfaces with R_a

The suction-based attachment force of AOS-sm is determined by ΔP_o, $D_{v,in}$, and a compensation factor (γ) according to pressure leakage, as shown in Equation (2). Among these factors, ΔP_{max} and $D_{v,in}$ depend on the structural changes of AOS-sm under pneumatic operation, whereas γ is related to interfacial interactions between the infundibulum of AOS-sm and the object;[2] γ increases as the interaction between the surface and the infundibulum of AOS-sm is weakened and decreases as the interaction is strengthened. This correlation can be interpreted based on the adhesive force data for each structure of the infundibulum according to the surface roughness. As shown in Figure S7, the dependence of the adhesive force on the roughness can be expressed using the following linear approximation:

$$r \approx \alpha R_a + 1, \tag{S3}$$

where r is ratio of the reciprocal adhesion forces ($r = \sigma_{flat}/\sigma_{Ra}$), σ_{flat} is the interaction force of infundibulum with a flat substrate, $\sigma_{Ra}$is the interaction force of the infundibulum with a rough substrate with R_a, and α is the leakage parameter determined by the induction between each infundibulum and the substrate surface. Assuming that the ratio of total suction-based attachment force of AOS-sm is proportional to the interaction force of the infundibulum,

$$\frac{F_{s,W,flat}}{F_{s,W,Ra}} \approx \frac{\sigma_{flat}}{\sigma_{Ra}} = r \approx \alpha R_a + 1, \tag{S4}$$

where $F_{s,W,flat}$ is the suction-based attachment force of AOS-sm on a flat substrate, and $F_{s,UW,Ra}$ is the suction-based attachment force on a surface with a roughness of R_a. As these forces can be expressed as $F_{s,W,flat} = -\Delta P_{max}(1-\gamma_{flat})\frac{\pi D_{v,in}^2}{4}$, $F_{s,W,Ra} = -\Delta P_{max}(1-\gamma_{Ra})\frac{\pi D_{v,in}^2}{4}$, this expression can be rewritten as follows:

$$\frac{-\Delta P_{max}(1-\gamma_{flat})\frac{\pi D_{v,in}^2}{4}}{-\Delta P_{max}(1-\gamma_{Ra})\frac{\pi D_{v,in}^2}{4}} \approx \frac{(1-\gamma_{flat})}{(1-\gamma_{Ra})} = \frac{\sigma_{flat}}{\sigma_{Ra}} = r \approx \alpha R_a + 1, \tag{S5}$$

where γ_{Ra} is the compensation factor on a rough substrate with R_a, α is the adaptability constant, R_a is the average roughness of the substrate, and γ_{flat} is the compensation factor on a flat substrate. This relationship can be expressed with respect to $(1-\gamma_{Ra})$ as follows:

$$(1-\gamma_{Ra}) \approx \frac{1-\gamma_{flat}}{\alpha R_a + 1}. \tag{S6}$$

Therefore, the suction-based attachment force of AOS-sm considering the suction-based attachment force due to structural changes ($\approx -\Delta P_0 \frac{\pi D_{v,in}^2}{4}$) and interfacial interactions ($\approx \frac{1-\gamma_{flat}}{\alpha R_a+1}$) between the infundibulum and the surface can be expressed as follows.

$$F_{s,W} \approx -\Delta P_0 \left(\frac{1-\gamma_{flat}}{\alpha R_a+1}\right)\frac{\pi D_{v,in}^2}{4} + \sigma_{rim,w}A' \tag{S7}$$

FEM Simulation

FEM simulation was performed using commercial software (COMSOL Multiphysics version 5.2a, ALTSOFT, Republic of Korea, license number: 5084832). Custom 2D models with sizes of 500 × 510 mm^2 were built. The model is automatically tangent to the tetrahedral surface. The stress and strain on the structure were analyzed using the structural mechanical module using the following equations:

$$\nabla \cdot \sigma + F = \rho, \tag{S8}$$

$$\varepsilon = \frac{1}{2}[\nabla u + (\nabla u)^T], \tag{S9}$$

$$\delta = C : \varepsilon, \tag{S10}$$

where u is the displacement of each point, σ is the infinitesimal strain tensor, C is the elasticity tensor, $\delta$is the stress tensor, and F is the external force. For simplicity, pseudostatic analysis and a linear elastic material model were used. The elastic modulus was set at 300 kPa and Poisson's ratio was set at 0.49 for this specific model.

Fabrication of Microdenticle Mold

As shown in Figure S1, silicon molds with microhole patterns of 50 μm and an aspect ratio (AR; width-to-depth ratio) of 1 were prepared by photolithography and subsequent reactive ion etching. The molds were treated with a fluorinated self-assembled monolayer (SAM) solution ((tridecafluoro-1,1,2,2-tetrahydrooctyl)-trichlorosilane; FOTCS, Gelest Corp, USA) diluted in 0.03 M of anhydrous heptane (Samchun Chemical, Republic of Korea) in an Ar atmosphere. Droplets of an polyurethane acrylate (s-PUA) prepolymer (PUA MINS 301 RM; Minuta Tech, Republic of Korea) droplets were dispensed onto the master, and a polyethylene terephthalate (PET) film (50 μm) was pressed lightly against the liquid droplets as a supporting backplane. Following this partial wetting of the prepolymer, air bubbles were trapped in the microhole chambers owing to the viscosity and interfacial tension of s-PUA.[3] After UV exposure, the s-PUA master was affixed to a glass substrate, and a polydimethylsiloxane (PDMS) mixture (Sylgard 184, Dow Corning, USA) with 10 wt% curing agent was spin-coated on top of s-PUA at 300 rpm for 1 min. After thermal curing at 70 °C for 2 h, the PDMS master was placed in a vacuum chamber (~10^{-1} Pa) with 1 mL of FOTCS. The chamber was heated at 50 °C for 2 h for vapor deposition of the SAM reagents onto the samples, followed by annealing at 80 °C for 30 min.

Characterization of Microdenticles and Substrate Roughness

To observe the morphologies of the microdenticles and rough substrates, scanning electron microscopy (SEM) images were collected using a field-emission scanning electron microscope (7600F, JOEL, Japan) at acceleration voltages of 5–15 kV and a working distance of 8.0 mm. The samples were coated with platinum before analysis to prevent electron charging. Optical microscopy (OM) images of the patch samples were collected using a light microscope (BX51, Japan). For each point, the vertical distance (y_i) from the mid-line of the surface step height corresponding to the horizontal displacement was measured and the average roughness was calculated using $R_a = \frac{1}{n}\sum_{i=1}^{n} y_i$.

Fabrication of Rough Substrates

To obtain s-PUA substrates with rough morphologies (R_a = 40, 80, 162, and 200 μm), a PDMS mixture with 10 wt% curing agent was poured onto sandpaper of varying roughness (FEPA Grit Designations P360, P180, P100, and P80, which correspond to average grain sizes of 40.5, 82, 162, and 201 μm, respectively) (see Figure 4d). After thermal curing at 70 °C for 2 h, the cured PDMS roughness mold was peeled off the sandpaper. Droplets of the s-PUA prepolymer were then dispersed evenly onto the PDMS mold and a PET film was pressed lightly against the liquid droplets as a supporting backplane. The s-PUA was exposed to UV light for 2 min, and the PET film with a rough s-PUA substrate was peeled off. The s-PUA replica was further exposed to UV light for 12 h to achieve complete curing (Figure S19).

Fabrication of Curved Substrates

Surfaces with various curvatures and a height of ~3 cm were designed using 3DCAD software. Upon fabrication, flat s-PUA substrates (area ~ 4 × 4 cm^2) were laminated onto the curved surfaces so that all measurements were performed using the same substrate materials (Figure S19). Herein, the curvature of the substrates is defined by the AOS-sm-to-surface curvature ratio (*r/R*), where *r/R* < 1.

Fabrication of Octo-Gripper

The 3D-printed molds for the octo-gripper arm were designed via CAD drawings. The molds were treated in a SAM solution of 1% octadecyltrichlorosilane(ODTS) in hexane for 1 h at ambient temperature and then heated in an oven for 12 h. Subsequently, the inner and outer

molds for the inflatable top portion were assembled, and the Dragon Skin 10 prepolymer was deposited within 3D-printed molds (Figure S16). After curing at room temperature for 4 h, the top portion was demolded from the assembly. A piece of paper, which served as the strain-restraining layer, was placed into the mold for the bottom portion before adding Dragon Skin 10. The top portion of the octo-gripper arm was positioned inside the bottom layer mold before curing, so that the prepolymer was cured in place. After the assembly was cured at room temperature for 4 h, three AOS-sms were arrayed onto the surface of the bottom portion of the octo-gripper arm and fixed using a silicone adhesive (Figure S16). Finally, curing at room temperature for 6 h produced the integrated octo-gripper.

Pressure and Signal Circuits for Actuation of AOS-sms and Octopus-Arm-Inspired Soft Actuator (OASA)

As shown in Figure S17, the pressure controller comprises a microprocessor, four metal oxide semiconductor field-effect transistors (MOSFETs), four high-speed solenoid valves (CH-1290; Versoix, Switzerland), and a compressor with a pressure chamber. The microprocessor selectively provides a “valve open” signal to the solenoid valve through the MOSFETs. A voltage signal of 5 V generated by the microprocessor is amplified to 12 V through the MOSFETs and applied to the solenoid valves. Meanwhile, the compressor generates pressure and stores it in the pressure chamber, which is transmitted to the AOS-sms and the OASA when the solenoid valve is selectively opened. The AOS-sms and the OASA are separately connected to two solenoid valves, which perform pneumatic actuation input and ventilation tasks.

Practical Demonstrations of Object Transfer Using AOS-sms and the OASA

A commercial open-hardware oriented platform (OpenMANIPULATOR-X RM-X52-TNM, ROBOTIS, Republic of Korea) was utilized to demonstrate the practical application of the AOS-sms and OASA to the highly adaptable attachment and transport of various objects. The AOS-sms and OASA were connected to the manipulator using a 3D-printed design, and a conventional Arduino board was used to transmit signals from a computer.

Transfer Demonstrations with Porcine Liver and Heart

Fresh porcine liver and heart (postmortem) organs were obtained from a local slaughterhouse (Jidong, Republic of Korea) and stored frozen. The organs were immersed in deionized water for 2 h followed by light drying under ambient condition. The organs were then

placed under water in a glass tub to initiate attachment and transport experiments with the OASA.

Supplementary Figures

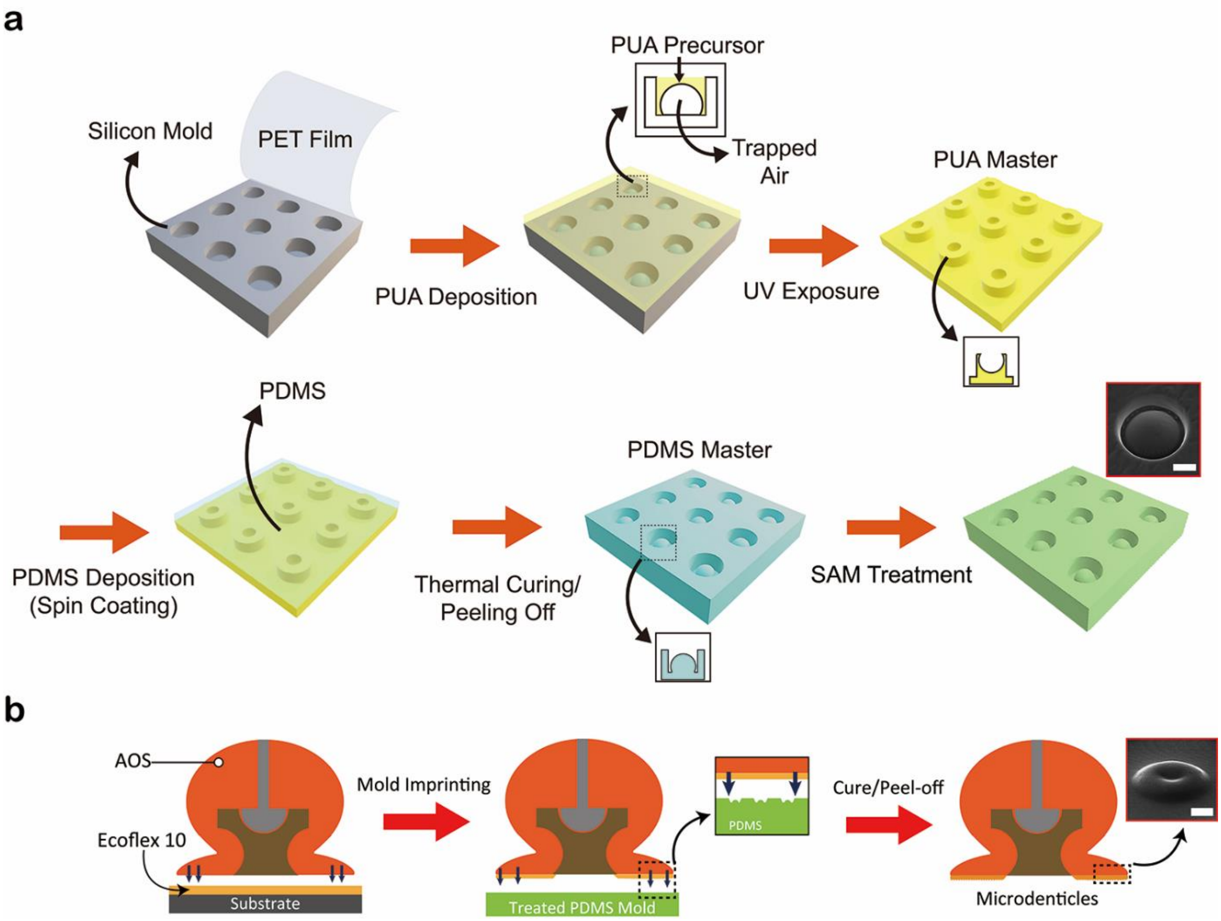

Figure S1. Fabrication of highly soft microdenticles. a) Schematic of the fabrication process for the microdenticle mold (inset: SEM image of a single architecture on the SAM-treated PDMS master; scale bar, 10 μm). b) Schematic of the microdenticle-imprinting process on the AOS-sm rim (inset: SEM image of a single microdenticle; scale bar, 10 μm).

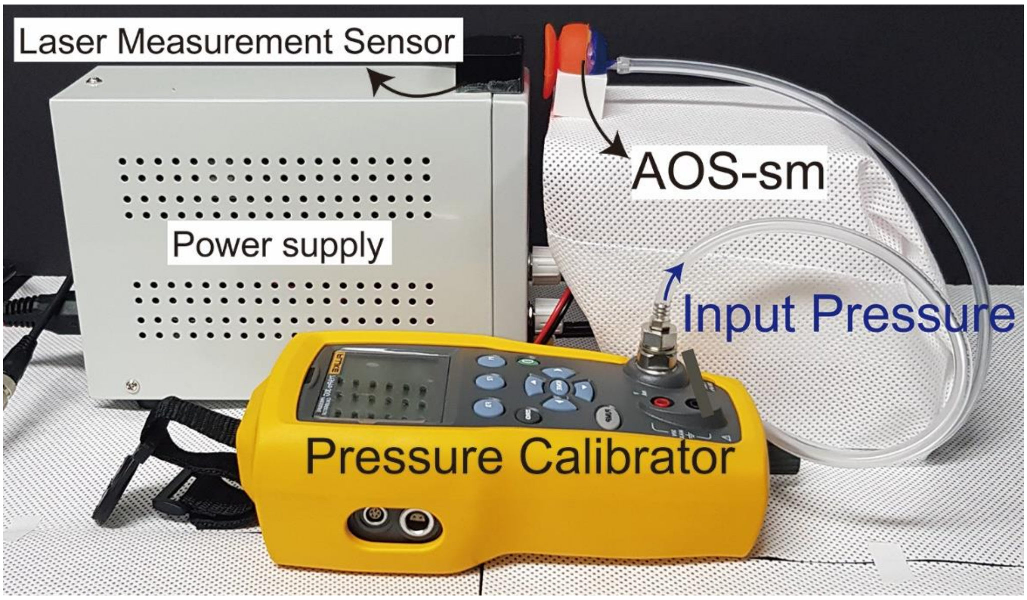

Figure S2. Experimental setup for AOS-sm geometry measurements during pneumatic actuation. Representative photograph of the experimental setup used to measure AOS-sm height changes.

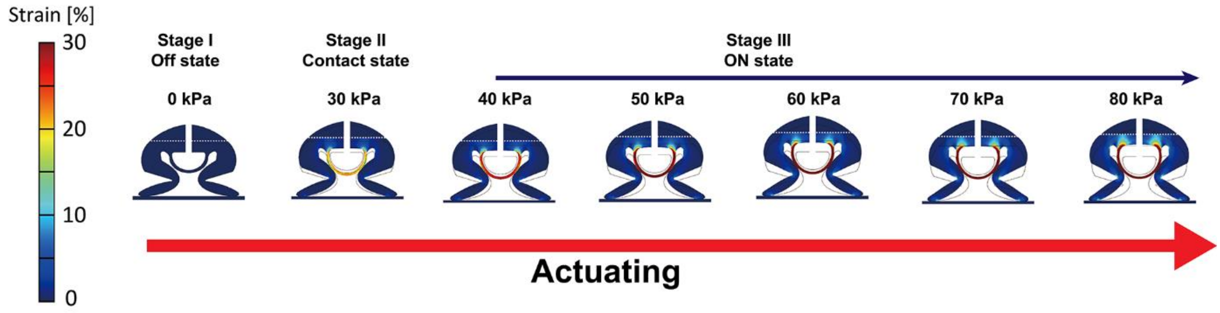

Figure S3. FEM simulation analysis of AOS-sm actuation.

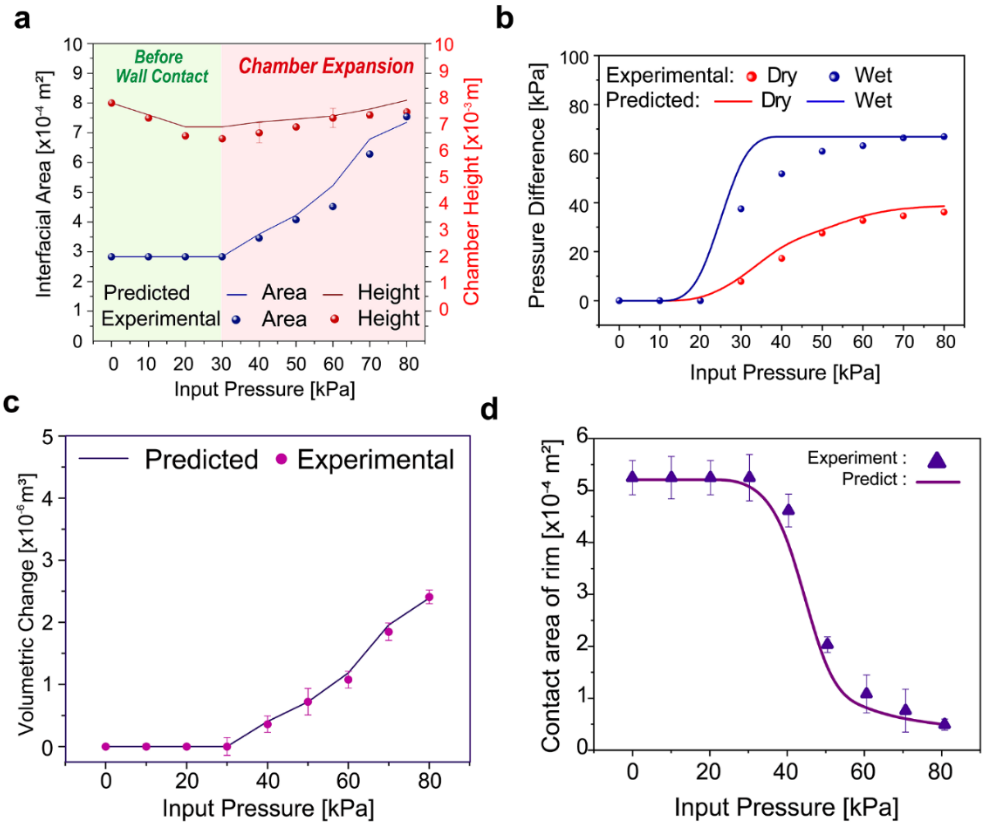

Figure S4. a) Predicted and experimental profiles of interfacial area and height corresponding to variances in pressure input during actuation in dry condition. b) Predicted and experimental pressure differences in AOS-sm with changes in pressure input. c) Predicted and experimental volumetric changes in AOS-sm with changes in pressure input. d) Predicted and experimental contact area of AOS-sm rim. Error bars represent standard deviations (n = 10). As the input pressure increases in the AOS-sm, the contact area at the interface of the microstructured rim decreases.

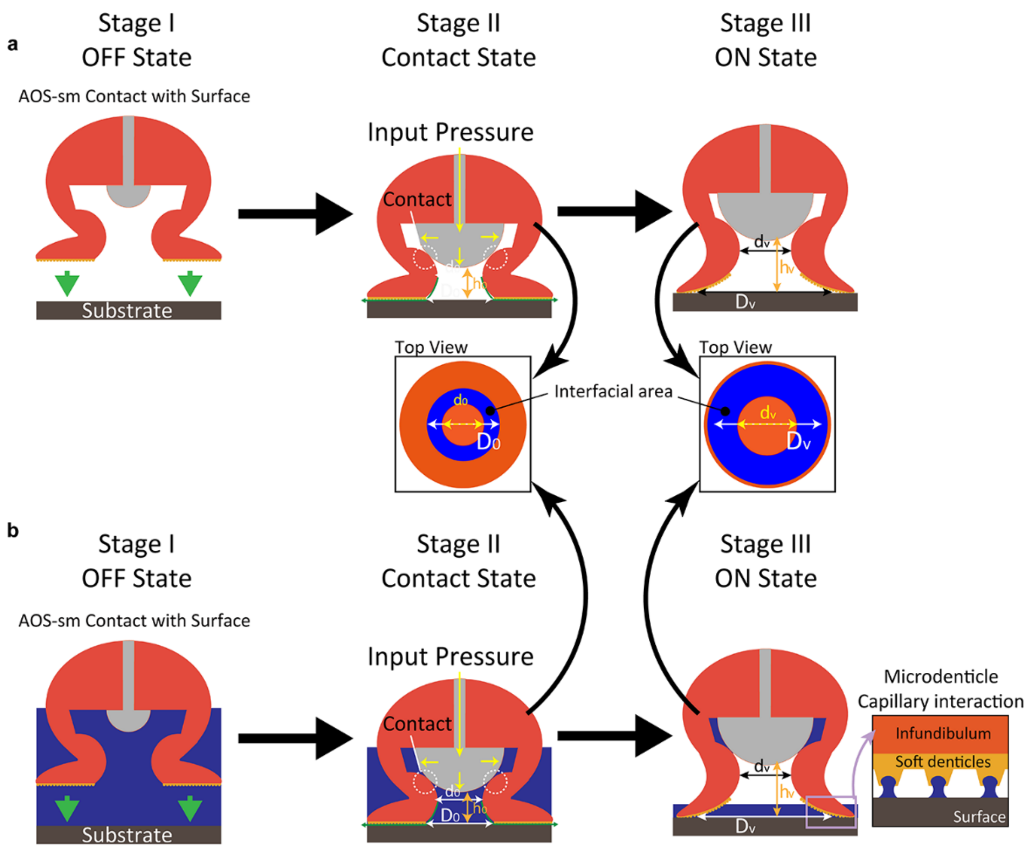

Figure S5. Schematic of the attachment mechanisms of AOS-sm under a) dry and b) wet conditions.

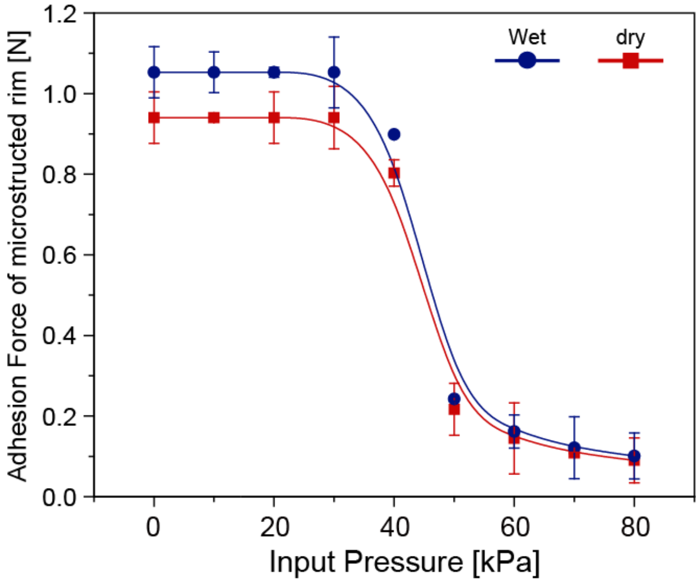

Figure S6. Adhesion force of microstructured rim of AOS-sm according to input pressure in dry and wet environment. Error bars represent standard deviations (n = 10). As the input pressure increases, the adhesion area of the interface decreases, so the contribution of the microstructured rim to the adhesion of AOS-sm decreases.

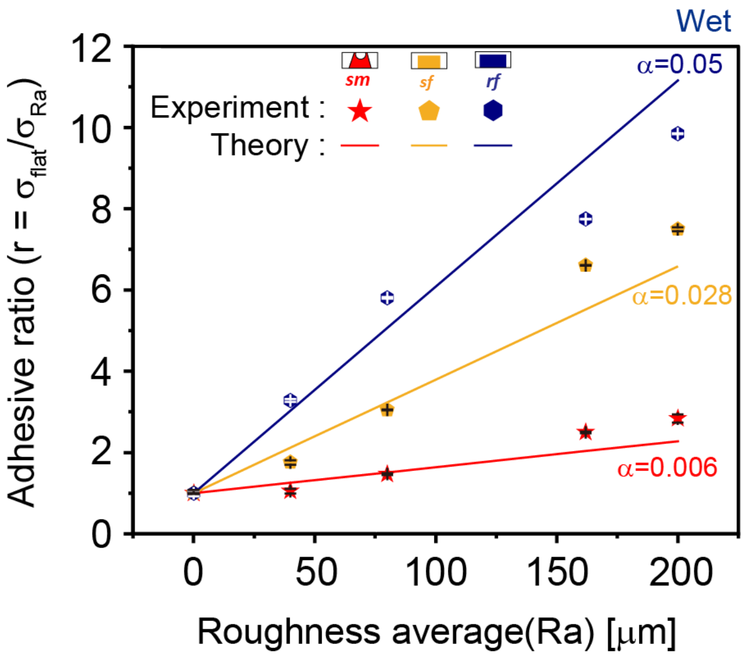

Figure S7. Adhesive ratios for rf (navy), sf (orange), and sm (red) on surfaces with varying roughnesses under water. Error bars represent standard deviations (n = 10).

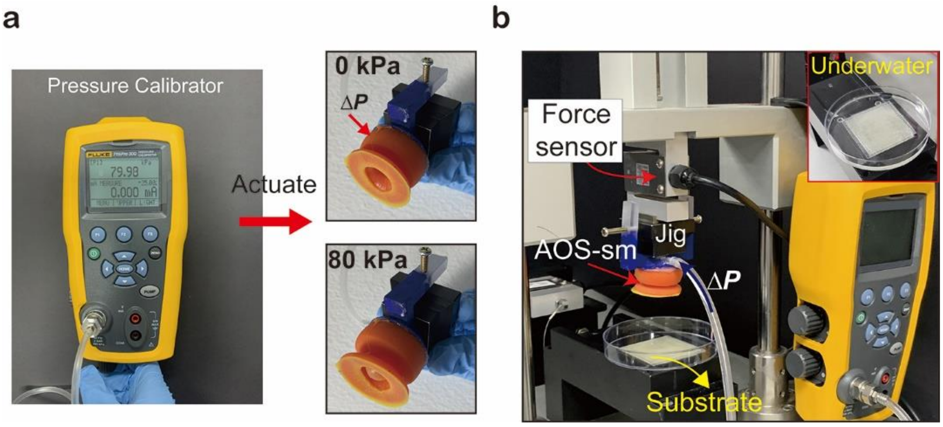

Figure S8. Experimental setup for measurements of dry and wet adhesion during pneumatic actuation. a) Photographs of the electric pressure calibrator and the AOS before and after pneumatic actuation. b) Pull-off adhesion measurement using custom-built equipment (inset: s-PUA substrate under wet conditions).

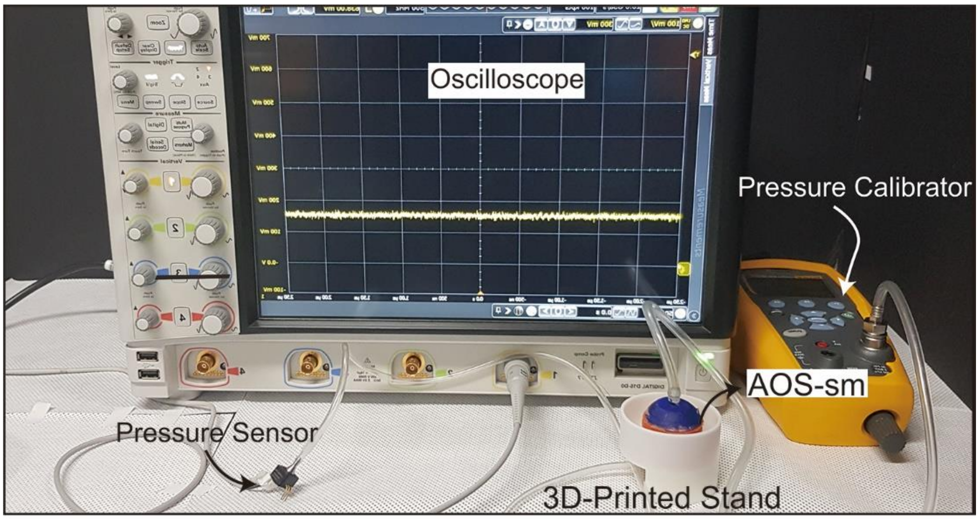

Figure S9. Experimental setup for measuring dry and wet pressure differences during pneumatic actuation.

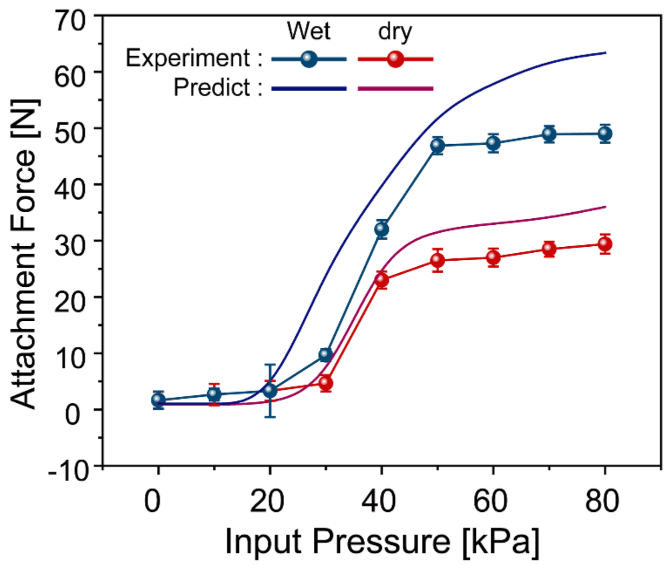

Figure S10. Predicted and experimental AOS-sm attachment force data according to input pressure in dry and wet environment. Error bars represent standard deviations (n = 10).

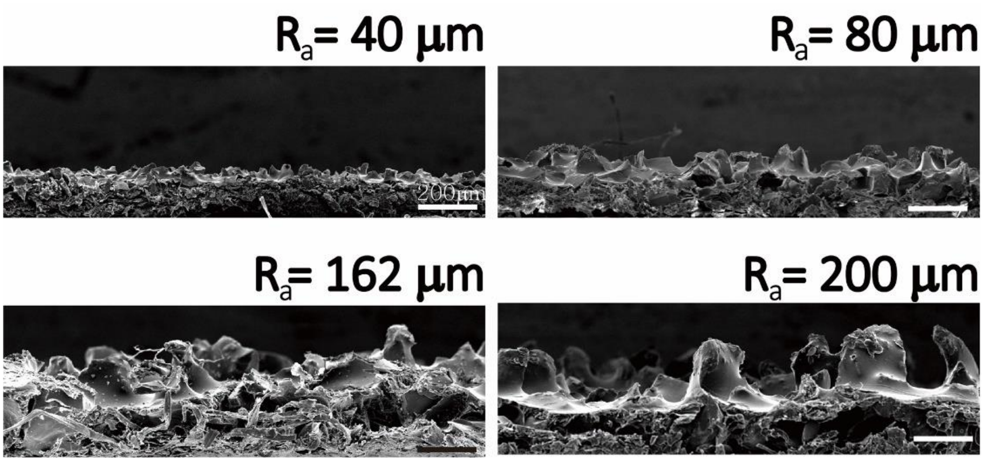

Figure S11. Cross-section sem image of various roughness (R_a = 40, 80, 162 and 200 μm)

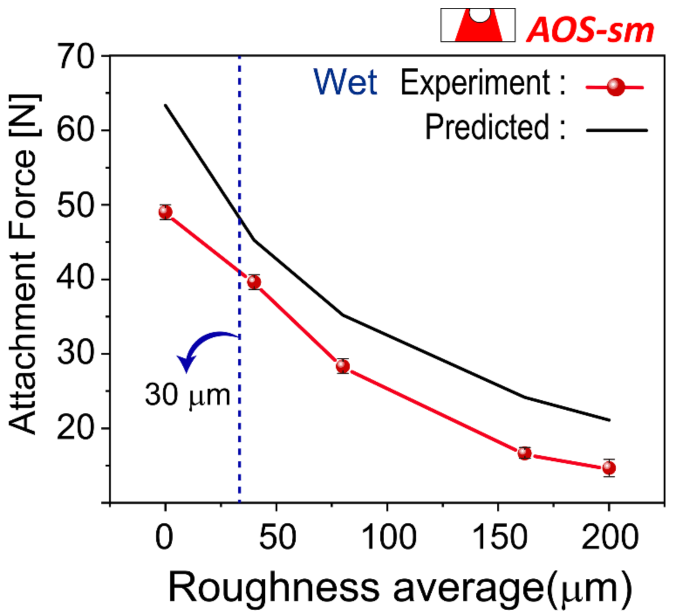

Figure S12. Predicted and experimental AOS-sm attachment force data according to surface roughness. The blue dot line Indicate the same R_a as the microdenticle size. Error bars represent standard deviations (n = 10).

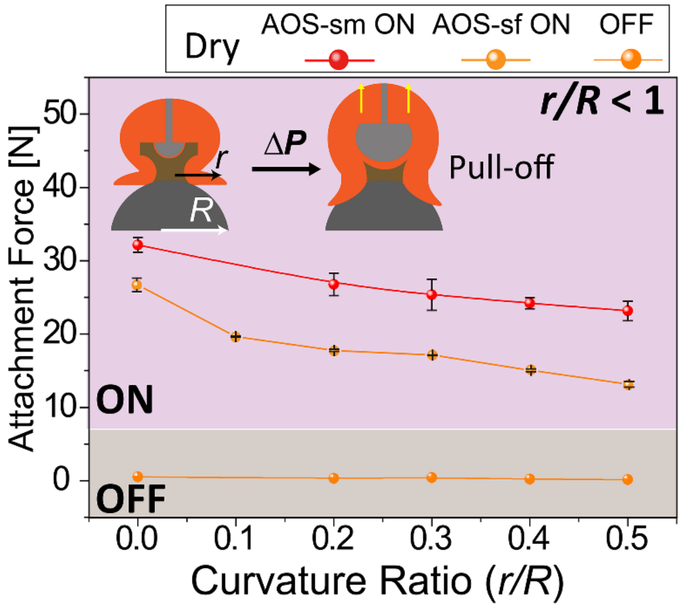

Figure S13. Adhesive strength profiles for on (80 kPa) and off (0 kPa) inputs of pneumatic pressure under dry conditions at various AOS-sm-to-surface curvature ratios (*r*/*R*). Error bars represent standard deviations (n = 10).

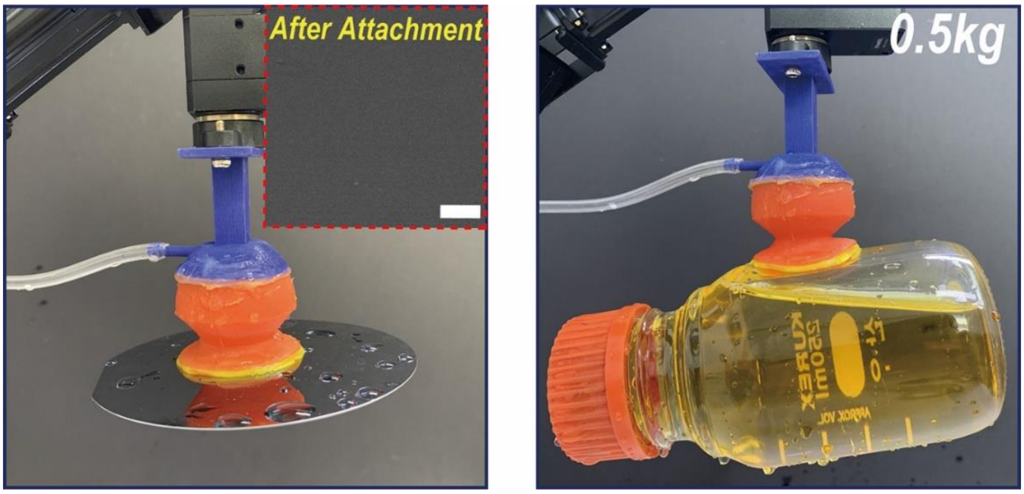

Figure S14. Photographs of the AOS-sm attached to a silicon wafer and a glass bottle of apple juice.

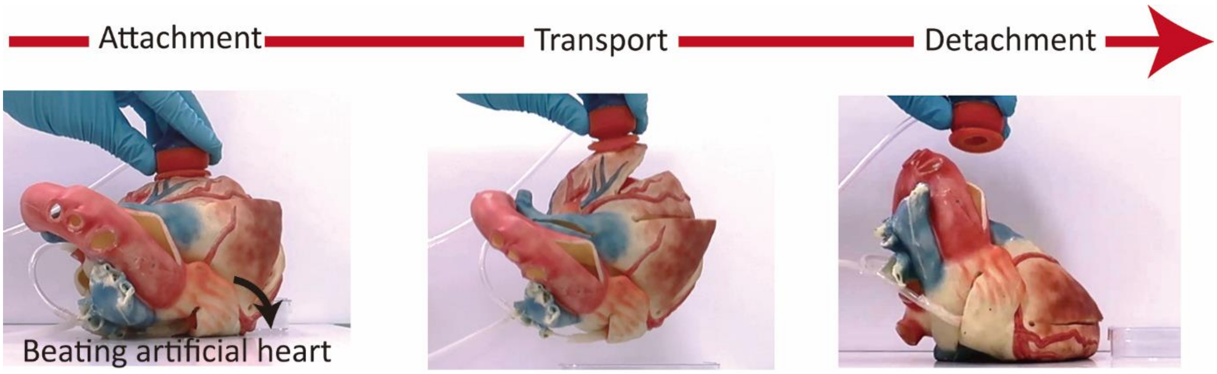

Figure S15. Photographs of the AOS-sm transport to a beating artificial heart.

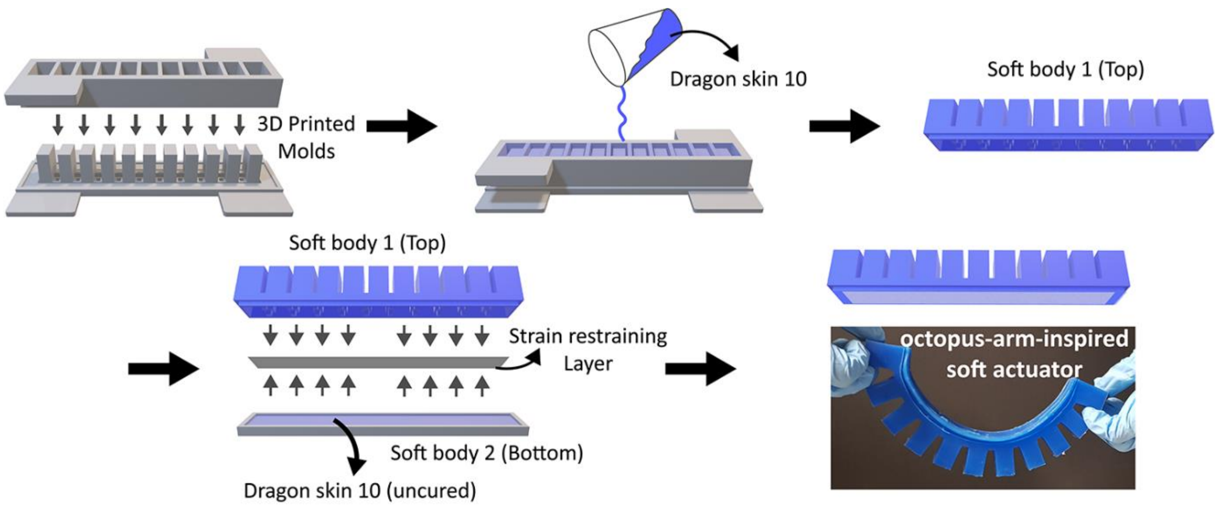

Figure S16. Schematic of the fabrication process for the octo-gripper arm using 3D-printed molds (inset: photograph of the OASA).

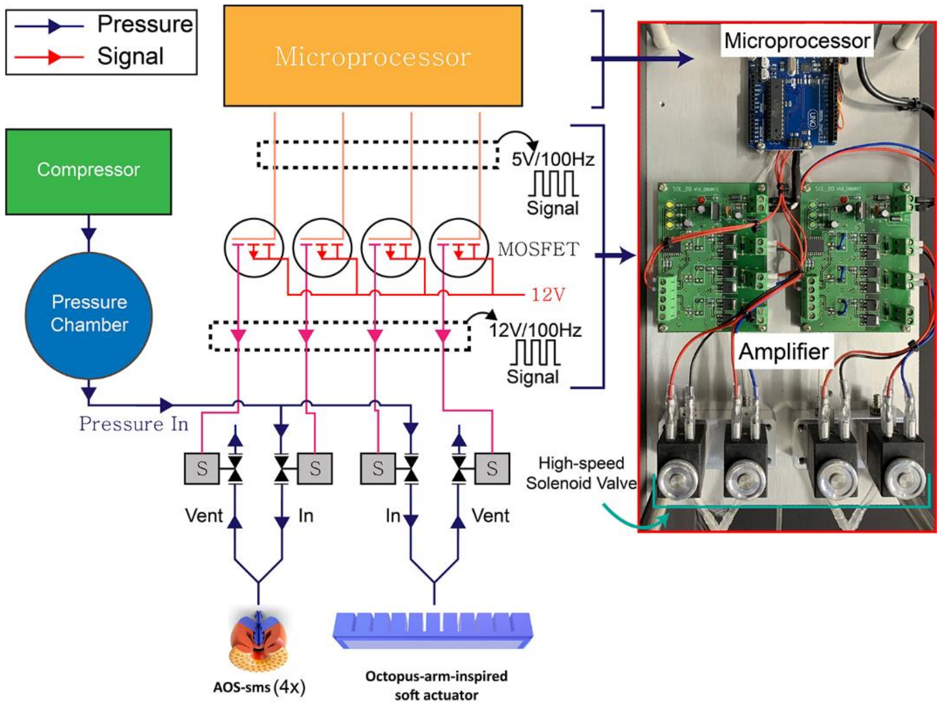

Figure S17. Schematic of pressure (blue) and signal (red) circuits for actuation of the AOS-sms and the OASA (inset: photograph of the designed circuit).

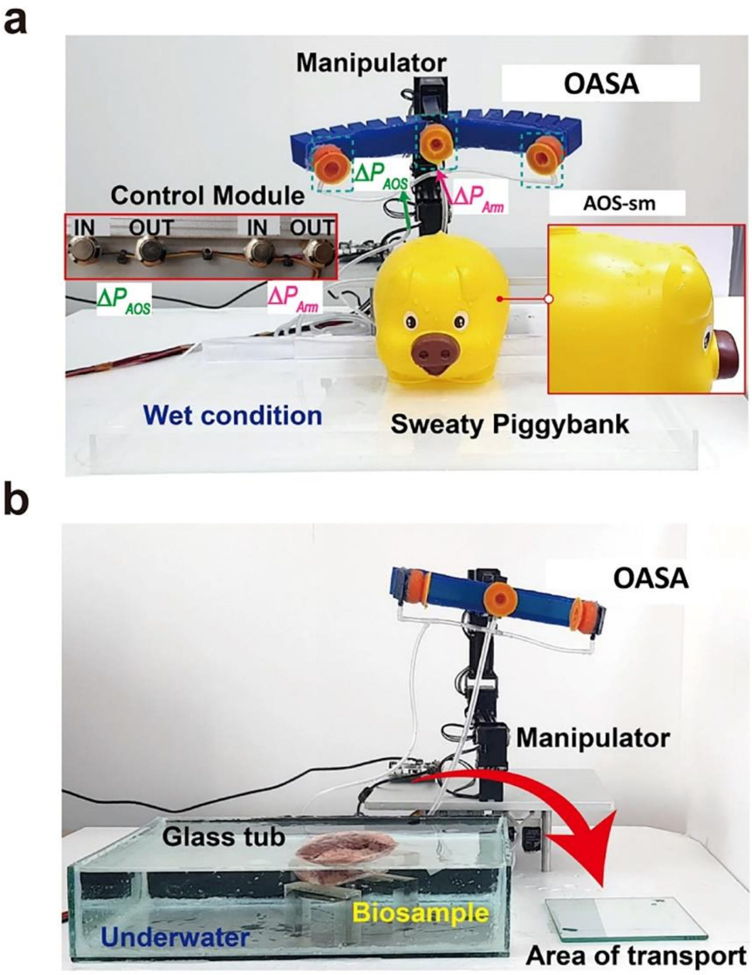

Figure S18. Transportation demonstrations using the OASA. a) Experimental setup of the OASA connected to the manipulator to handle a sweaty piggy bank (left inset: control module for input/output of pneumatic pressure for both the AOS-sms and the OASA; right inset: sweaty condition of the piggy bank). b) Experimental setup of the OASA for transportation of biosamples under wet conditions.

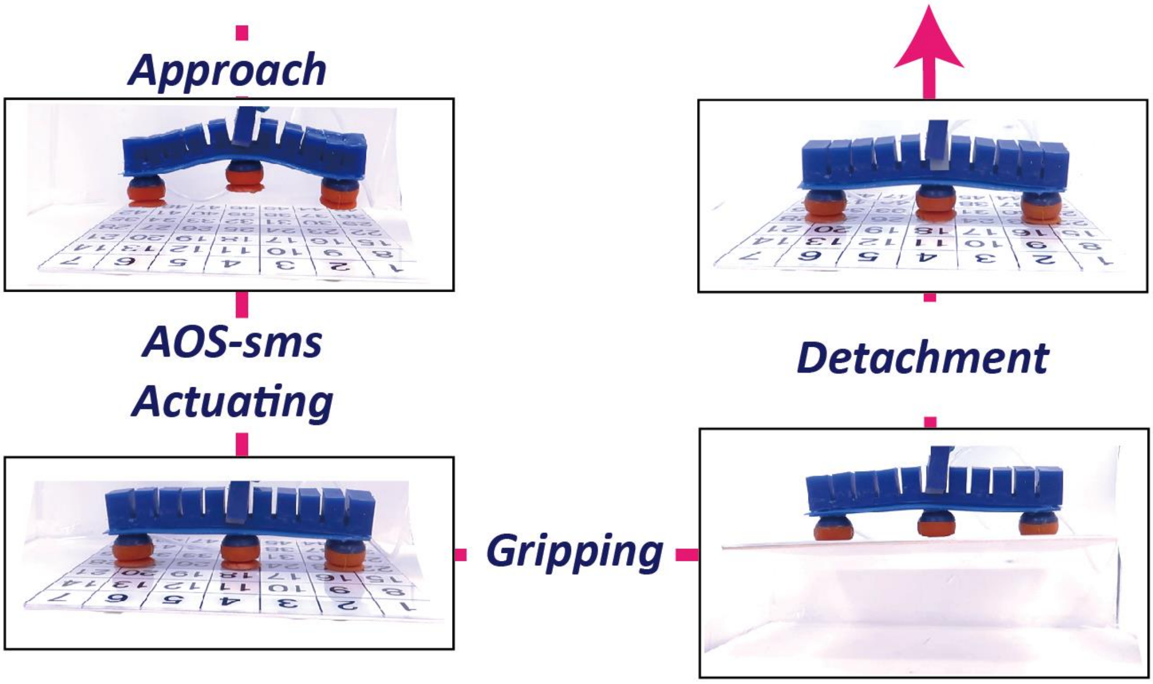

Figure S19. Photographs of the OASA with AOS-sms transport to a bigger dimensions object.

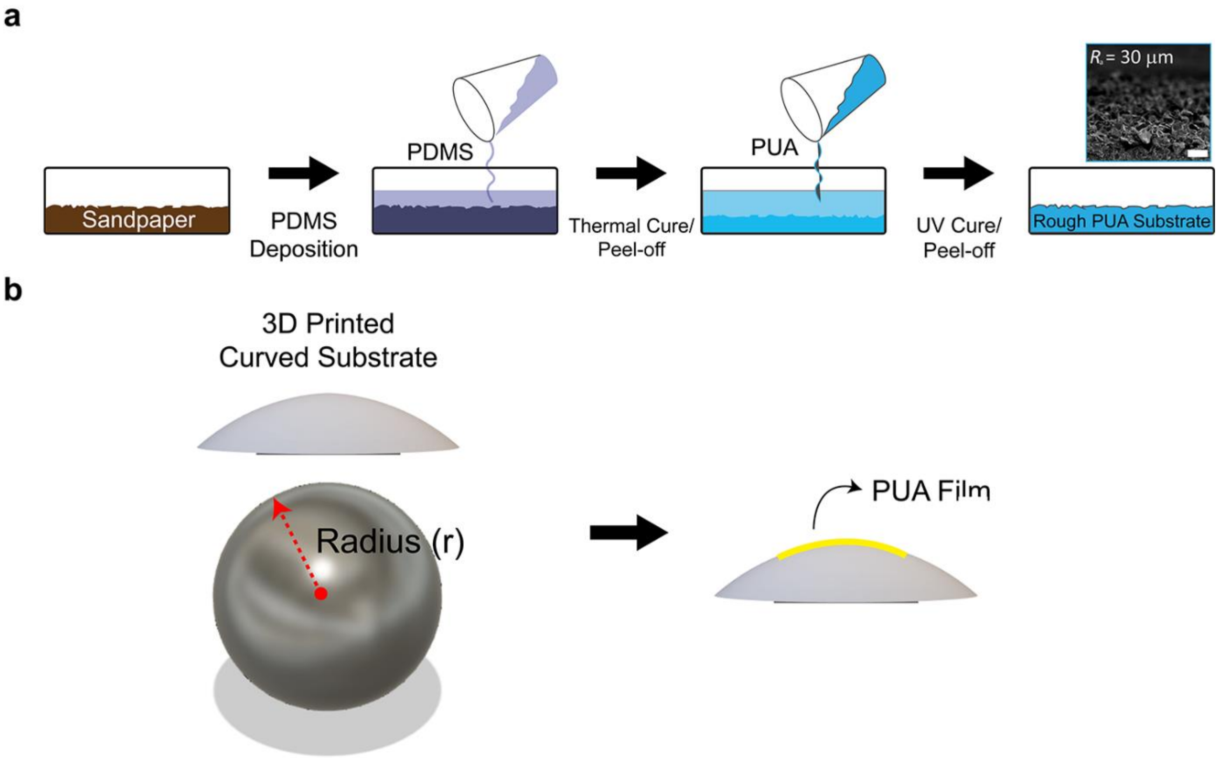

Figure S20. Fabrication methods for a) rough s-PUA and b) curved substrates.

Architecture	Materials	Adhesive in dry environment	Adhesive in wet environment	Actuation method	[ref]
**Gecko**	Polyutethane	0.324 N/cm^2	-	Pneumatic	[4a]
	PDMS	0.057 N/cm^2	-	UV light	[4b]
	PDMS/Ecoflex	1.1 N/cm^2	-	Pneumatic	[4c]
**Tree frog**	Dragon skin/Ecoflex	0.145 N/cm^2	-	Pneumatic	[4d]
**Bloodworm**	Silicone	2.079 N/cm^2	-	Pneumatic	[4e]
**Suckerfish**	Mold star/Ecoflex		5.375 N/cm^2	Elctrostatic	[4f]
	Dragon skin		0.243 N/cm^2	Pneumatic	[4g]
**Octopus**	PDMS	5 N/cm^2	4.9 N/cm^2	Elctrothermal	[4h]
	Dragon skin/Ecoflex	3.23 N/cm^2	5.6 N/cm^2	Pneumatic	[4i]
	PDMS	1.43 N/cm^2	-	Magnetic	[4j]
	Silicone	-	2.526 N/cm^2	Pneumatic	[4k]
	Mold star	0.131 N/cm^2	-	Pneumatic	[4l]
**Our work**	Dragon skin/Ecoflex	3.6 N/cm^2	6.098 N/cm^2	Pneumatic	

Table S1. Adhesive performances of previously-developed bioinspired adhesive actuators in dry/wet environments and comparison to our work.

REFERENCES

[1] a) H. J. Lee, S. Baik, G. W. Hwang, J. H. Song, D. W. Kim, B.-y. Park, H. Min, J. K. Kim, J.-s. Koh, T.-H. Yang, *ACS nano* 2021, **15**, 14137; b) Del Campo, A., & Arzt, E, Macromolecular bioscience, 2007, **7**(2), 118-127.
[2] B. Persson, C. Yang, *Journal of Physics: Condensed Matter* 2008, **20**, 315011.
[3] S. Baik, D. W. Kim, Y. Park, T.-J. Lee, S. Ho Bhang, C. Pang, *Nature* 2017, **546**, 396
[4] a) S. Song, M. Sitti, *Adv. Mater.* 2014, **26**, 4901; b) E. Kizilkan, J. Strueben, A. Staubitz, S. N. Gorb, *Sci. Rob.* 2017; c) S. Song, D. M. Drotlef, D. Son, A. Koivikko, M. Sitti, *Adv. Sci.* 2021, **8**, 2100641; d) Sandoval, J. A., Xu, T., Adibnazari, I., Deheyn, D. D., & Tolley, M. T. IEEE Robotics and Automation Letters, 2022, 7(2), 4134-4141; e) D. Sui, Y. Zhu, S. Zhao, T. Wang, S. K. Agrawal, H. Zhang, J. Zhao, *Soft Rob.* 2022, **9**, 36; f) S. Su, S. Wang, L. Li, Z. Xie, F. Hao, J. Xu, S. Wang, J. Guan, L. Wen, *Matter* 2020, **2**, 1207 g) Y. Wang, X. Yang, Y. Chen, D. K. Wainwright, C. P. Kenaley, Z. Gong, Z. Liu, H. Liu, J. Guan, T. Wang, *Sci. Rob.* 2017, **2**, eaan8072; h) S. Baik, G. W. Hwang, S. Jang, S. Jeong, K. H. Kim, T.-H. Yang, C. Pang, ACS Appl. Mater. Interfaces 2021, 13, 6930; i) H. J. Lee, S. Baik, G. W. Hwang, J. H. Song, D. W. Kim, B.-y. Park, H. Min, J. K. Kim, J.-s. Koh, T.-H. Yang, *ACS nano* 2021, **15**, 14137; j) S. Wang, H. Luo, C. Linghu, J. Song, *Adv. Funct. Mater.* 2021, **31**, 2009217; k) M. Wu, X. Zheng, R. Liu, N. Hou, W. H. Afridi, R. H. Afridi, X. Guo, J. Wu, C. Wang, G. Xie, *Adv. Sci.* 2022, 2104382; l) Z. Xie, A. G. Domel, N. An, C. Green, Z. Gong, T. Wang, E. M. Knubben, J. C. Weaver, K. Bertoldi, L. Wen, *Soft Rob.* 2020, **7**, 639.
